# Staff perspectives on independence and collaborative practices in the IRIS intervention: an evaluation in Swedish LSS residential homes

**DOI:** 10.3389/fresc.2025.1571233

**Published:** 2025-05-12

**Authors:** Sylvia Olsson, Carina Loeb, Munir Dag

**Affiliations:** ^1^ School of Health, Care and Social Welfare, Mälardalen University, Eskilstuna, Sweden; ^2^ Department of Behavioral, Social, and Legal Sciences, Örebro University, Örebro, Sweden

**Keywords:** intellectual disability, disability, interventions, self-efficacy, inclusion

## Abstract

**Background and purpose:**

Intensive Rehabilitation Coordination (IRIS), is an intensive, health-promoting habilitative intervention designed to prevent, reduce, and compensate for individuals’ support needs while enhancing their independence. The purpose of this study is to explore staff experiences with the IRIS intervention in a municipality's LSS^1^ residential homes, with a focus on how independence is defined and operationalized and the resulting impact on service delivery.

**Methods:**

A qualitative study was conducted in which staff from various LSS residential homes were interviewed. The interviews were structured to examine differences in the interpretation of independence, the strategies used to support service users, and the collaborative nature of the intervention. Participants were asked to describe their approaches to encouraging or supporting independence in daily tasks and their interactions with quality coordinators.

**Results:**

The analysis revealed a lack of a shared definition of independence among staff, leading to varied approaches in supporting service users. Some staff members actively promoted independence by encouraging service users to perform everyday tasks on their own, while others performed these tasks for the service users due to practical considerations such as convenience and efficiency. Despite these variations, staff reported that service users still have the opportunity to make their own decisions regarding daily activities. Overall, the IRIS intervention was viewed positively, with its collaborative implementation cited as a key strength.

**Discussion:**

The findings suggest that the absence of a unified conceptualization of independence can lead to inconsistent practices in LSS residential homes, potentially affecting the effectiveness of the intervention. The collaborative approach of IRIS, which fosters joint efforts among municipal professionals, appears to be instrumental in understanding and addressing service users’ needs. Future research should focus on establishing a more standardized definition of independence to improve intervention consistency and outcomes for service users.

## 1 Introduction

Intensive Rehabilitation Coordination (IRIS, in Swedish: Intensiv Rehabiliterande InsatsSamordning) is a form of health-promoting and habilitative intervention that aims to prevent, reduce, and compensate for an individual's support needs. IRIS is an intervention used by staff to assist and support individuals with intellectual disabilities in achieving a more independent life. The intervention aims to support individuals with disabilities in becoming as independent as possible and in strengthening their confidence in their own abilities (self-efficacy), rather than serving as a means for organizational efficiency gain. IRIS involves collaboration between various professionals, including occupational therapists, physiotherapists, nurses, and care managers. IRIS also aims to increase the individual's independence and sense of empowerment. The intervention is aimed at people who have been granted access to special housing, residential services, or supported living arrangements.

The present study focuses on IRIS interventions for individuals with intellectual disabilities. People with intellectual disabilities live in everyday circumstances that differ significantly from those of the general population. Due to the nature of their disability and the fact that they often have additional impairments, they often rely on societal support to manage their everyday lives. People with intellectual disabilities typically come into contact with a variety of professional services at an early stage, and often receive long-term, sometimes lifelong, support. As a result, they live in environments that are largely created and controlled by others (1–3).

The aim of this study is to explore the staff's experiences of IRIS as a health-promoting and habilitative intervention that has the goal of preventing, reducing, and compensating for the support needs of individual service users, as well as increasing their independence.

The research questions of the study are: (1) What do independence and increased autonomy mean to staff working with the IRIS intervention? (2) How can IRIS provide opportunities for individuals with intellectual disabilities to experience greater autonomy and independence in various life situations?

Throughout history, disabled people have often been excluded, marginalised, and discriminated against (4). In recent years, considerable efforts have been made in Sweden to adapt the physical environment, education system, and labour market to enable individuals with disabilities to participate in various spheres of life. These political and societal efforts to improve the living conditions of disabled people are aligned with the principle of normalisation. The core idea of the normalisation principle is that people with intellectual disabilities, as well as those with other disabilities, and members of other marginalised groups, should be offered what is considered “normal” by society, with the aim of facilitating the greatest possible independence (5). The normalisation principle emphasises that individuals' own demands, wishes, and choices should be taken into account as far as possible.

The normalisation principle focuses on eight aspects: a normal daily rhythm, a normal weekly rhythm, a normal yearly rhythm, normal developmental phases, normal self-determination, normal sexual patterns, normal economic patterns, and normal building standards. Nirje argues that the normalisation principle can support all people with disabilities, not just those with intellectual disabilities, as originally intended (5).

Despite various societal efforts, knowledge of disability theory remains low, according to Goodley, Hughes and Davis (6). The authors suggest that one reason for this is the low status of disability-related issues, despite legislative interventions. Disabled people continue to struggle for their basic rights and needs, such as healthcare, education, and employment opportunities. The social model gained significant traction with the publication of *Fundamental Principles of Disability* in 1976 (7). This manifesto was written by the Union of the Physically Impaired Against Segregation (UPIAS), an organisation that directly addressed the concerns of people with disabilities.

In our view, it is society which disables physically impaired people. Disability is something imposed on top of our impairments by the way we are unnecessarily isolated and excluded from full participation in society. [(7) p. 43].

The social model illustrates how various societal barriers negatively impact people with disabilities. Factors such as negative attitudes towards people with disabilities, poorly adapted physical environments, and structural discrimination all contribute to excluding people with disabilities—especially those with intellectual disabilities—from active participation in different areas of society. An inaccessible society, according to the social model, constitutes a form of systematic oppression, especially when organisations and public authorities frame accessibility issues as individual problems that disabled people are responsible for solving (7).

The factors that can affect the ability of disabled people to participate in different spheres of society can be viewed as either individual or societal (8, 9). Individual factors include the person's own resources, such as the degree of disability, self-efficacy, level of education, work experience, attitudes, motivation, and willingness to participate in various aspects of society (9, 10). Societal factors, on the other hand, include factors such as the physical environment, prevailing societal attitudes towards people with disabilities, and rules and regulations (8, 9, 11–14). Both individual and societal factors can act as resources or barriers for individuals attempting to participate in various areas of society.

Some societal factors that function as barriers may be linked to structural discrimination, which exists in Sweden as well as in other societies. Structural discrimination refers to the systematic negative treatment of certain groups of people (e.g., individuals with disabilities) when they attempt to participate in society, because of prevailing rules, regulations, norms, and power structures (15). For instance, from a global perspective, individuals with disabilities are to some extent excluded from participation in the labour market and thus from integration into mainstream society through employment (16–19).

In a study of young and adult individuals with intellectual disabilities in Sweden, Tideman (3) found that they experience poorer health, lower educational attainment, worse financial situations, and fewer social contacts than the general population. Most have little control over their lives and daily routines. Previous research indicates that the situation is similar in other Nordic countries. For instance, a study by Vedeler and Mossige (20, 21) shows that disabled people in Norway are treated as if they are not competent and are expected to settle for early retirement rather than ordinary jobs.

Independence and are central issues for people with disabilities. Söder (22) defines two distinct meanings of independence. The first is that individuals with disabilities should be able to perform various tasks and daily activities independently, without assistance from others. A person with a disability can, with the aid of various technological tools such as smart phones, computers, and eating aids, carry out tasks independently. Technological advancements have contributed to a more independent life, allowing individuals with disabilities to complete tasks without external help. The second meaning of independence is that people with disabilities should also have the opportunity to influence their situation and make own decisions, even if they are not able to perform all daily tasks independently. Söder does not distinguish between independence and autonomy; however, his second definition of independence can be interpreted as corresponding to the concept of autonomy. Here too, it is not primarily the degree of disability that determines the possibility to live independently, but rather the extent to which the environment is adapted and inclusive. Another author, Barron (23), highlights various barriers that individuals with disabilities face in their pursuit of independent living. One of these is the power of professionals, which can be manifested in professionals (assistants, caregivers) defining problems and solving them on behalf of the individual. Another barrier is external obstacles, such as inadequate physical adaptation of the surrounding environment. Jacobsson (24) argues that all individuals are to some extent dependent on each other. However, individuals with disabilities who require assistance in daily life may experience lifelong dependence on help and support. According to Jacobsson, this dependence can create a sense of powerlessness and violation of one's personal integrity.

Like Jacobsson (24) Spicker (25) and Eriksson (26) emphasise that people with disabilities need support and assistance to safeguard their rights and to live independent lives (25). According to Bandura, self-efficacy refers to believing in your own ability to manage different situations in your life—and there is strong empirical evidence that self-efficacy contributes to the experience of being able to control and influence important aspects of one's own life, thus enabling independence. Professionals working with individuals with disabilities play a key role in supporting the development of self-efficacy by facilitating positive experiences and providing encouraging social support, two of the most influential sources of self-efficacy according to Bandura's theory (27, 28). This approach aligns well with the principles of IRIS interventions.

## 2 Method

The chosen method was a qualitative study involving seven staff members (four women and three men) out of a total of 34 who had implemented IRIS, including support educators and support assistants. The educational backgrounds of the staff varied. One was trained as a social pedagogue, one held a master's degree in public health, one was trained as an assistant nurse, and one had begun studies in behavioural science and the Innovations Programme with a focus on working life. All participants had received training, either through their work or individually, in areas such as the Swedish Pedagogical Approaches and Methods (PFA) (29) and Mental Health First Aid (MHFA), and evidence-based psychology. The participants’ professional experience ranged from eight to 40 years.

### 2.1 Implementation

The selection consisted of staff from LSS^1^ residential homes in a medium-sized municipality in Sweden who had come into contact with the IRIS intervention. A total of 34 staff members were given the opportunity to begin working with IRIS. The Health and Social Care Department in Eskilstuna municipality contacted staff at various group homes to inform them about the study on IRIS. Those who were willing to participate were then invited to contact the researchers to schedule an interview. In total, seven staff members expressed interest in participating in the study.

All interviews were conducted at the participants' workplaces. With informed consent, the interviews were audio-recorded using a digital voice recorder. Each interview lasted between 45 and 90 min. The collected material was transcribed immediately after each interview.

The analysis of the collected data was guided by hermeneutic principles. Coding was carried out in several stages, beginning with open coding of the interviews and followed by a more systematic process. The categories that emerged during this process provided the foundation for structuring the study's results. Both the study's theoretical framework and previous research were used to inform the coding process.

After all interviews were transcribed, the data were read through in their entirety. During this initial reading, reflections on the interview situations and preliminary interpretations of participants' statements were noted. In parallel, a brief profile of each interviewee was compiled, including information such as age, gender, and educational background.

A second reading of the material was then conducted prior to the categorization into thematic areas, which were derived from the study's aims and research questions. During this rereading, the material was interrogated using guiding questions such as: How is the concept of independence defined? Is there a shared definition within the workplace? In what ways is this shared understanding communicated?

The next step in the analysis involved identifying a structure that corresponded to the content of the participants’ statements. The interview transcripts were read multiple times in order to identify themes, categories, patterns, similarities, and differences. Attention was also given to answers that addressed new research questions—questions that had not initially been posed, but which emerged during the analysis.

Gradually, a number of main themes and sub-themes were developed and organized into hierarchical levels. Under each theme, relevant quotations were compiled, each accompanied by a page reference to the original interview transcript. This allowed us to return to the source material and examine the quotations in their full context when needed.

### 2.2 Ethical considerations

The principles of ethical research were of particular importance, as the study involved people who in some cases were in a position of dependence. Prior to the project, an ethical review was conducted, which was approved on 30 March 2022 (Reference number 2022-01680-01). Good research practice (Swedish Research Council, 2011) was consistently applied throughout the project, and the ethical principles of the Swedish Research Council (2002) were followed. All participants were informed both orally and in writing about the purpose of the research and the methods that would be used. They were also informed that their participation was voluntary and that they could withdraw from the study at any time without explanation and without affecting their future relationship with the service.

## 3 Results

### 3.1 The concept of independence

Staff members defined the concept of independence in slightly different ways. Some defined it as being able to manage on one's own. In this context, staff focused on various life situations that a service user might encounter, for example, the service user's ability to independently book a dentist's appointment, purchase ingredients for a meal, and organise and manage their daily life.

Being able to take charge of and lead one's daily life, to independently decide how to manage various activities … Independence is really about handling the day-to-day without needing someone constantly behind you to guide, support, or assist you. (Staff member 5)

The above quote shows that some staff members defined independence as the service user's ability to carry out various daily tasks independently, without assistance from others.

Some staff members expressed that independence is about the right to make decisions about one's life in various ways, to make personal choices, to set boundaries and to speak up. “I think it's about having control over your own life, that you can really influence everything, that you have the opportunity to influence, to feel that you can make those choices in life” (Staff member 7). In the above quote, the staff emphasise the value of service users being able to make their own choices, just as people without disabilities do.

For another staff member, independence means having self-awareness in various situations. The interviewee provides an example of a service user preparing for a trip. In this case, independence involves the service user having the self-awareness to recognise that the trip (the situation) requires bringing one's medication. The interviewee adds another dimension to the concept of independence, suggesting that it also involves feeling heard by those around one.

Independence, to me, is when someone has self-awareness … about the situation around them … Like, I can travel to Gothenburg and bring my medication, knowing that I'll manage it at the right time … and also feeling heard, like when you say, “I”m in pain here', and you're acknowledged for that—this is how I would experience it or express it. (Staff member 4)

Another definition that emerges from the results is linked to the welfare system. According to Staff member 6, independence means not relying on financial assistance, having one's own apartment, and not needing medication.

Independence, for me, is not living on financial assistance, having my own apartment, and being able to do what I want without being tied to medication. That's independence to me … (Staff member 6)

Staff member 6's statement differs from those of the other interviewees in that independence in this context is linked to financial self-sufficiency. Staff member 6 suggests that independence means not living on benefits, implying that independent people have a job and a sufficient income to support themselves.

The results show that none of the group homes have a shared definition of the concept of independence that the staff can rely on. One of the interviewees mentioned that independence had been discussed at work: “Yes, well, we try, we have care plans and so on (Staff member 2)”. The results indicate that staff may have different definitions of independence, partly because there does not appear to be any regular or ongoing discussion in the workplace about how everyone should aim to support independent living.

### 3.2 The service user's ability to make personal choices as part of independence

All but one of the participating staff members stated that the service users have the opportunity to make their own choices about various daily tasks. Several provided simple examples related to mealtime, explaining that the service users can choose what they want to eat. Service users are typically offered two options, specifically as a strategy to avoid refusal by the user. Staff member 1 gave an example involving room cleaning. In such cases, the service user is given the option to choose between two times for the cleaning to take place, rather than choosing whether or not to have the cleaning done.

They always have the opportunity to make their own choices. I can give an example of a service user who lives here. We always give the service user two options to choose from … for example, if we have a cleaning task and it needs to be done. We clean three days a week, and it has to be completed. So, we can't ask, “Do you want cleaning today?” If the service user says no, then there won't be any cleaning. Instead, we have to ask the question in a slightly leading way, like, “Do you want us to clean at 10 o”clock or 1 o'clock?’ That way, they still have the opportunity to make a choice. (Staff member 1)

The staff member's statement above indicates that service users are given choices from the options that the staff themselves propose, rather than being able to make a free choice or opt out of the suggested alternatives. This is explained by the fact that certain tasks must be completed. Staff member 1 uses the example of cleaning, explaining that the service user's room must be cleaned three times a week. The service user cannot choose to skip the cleaning, but can choose between two times suggested by the staff. In this way, the staff can both ensure that their targets are met and complete a task that is important to the service user's daily life.

The interviews reveal that one staff member also highlighted staffing resources as a reason for restricting the service users' options.

Sometimes there's a staff shortage, and then you can't say, like, “Oh, I’d like to go to Stockholm”—no, unfortunately, that's not possible. But it has to do with the budget, or rather, with the way the service operates. We have a service user who loves to go to all the dances and events, but they can't go alone. The service users must have staff who come along … And if only one service user wants to go, we can't just send one staff member, because then there would only be two left here. It's impossible. (Staff member 3)

The interview material reveals that only one staff member believed that service users have the right to make choices and that the staff must respect those choices, even if the service user's decision would prevent an intervention from taking place.

Yes, I think so. We work a lot with needs-based work and free will. The right to self-determination carries a lot of weight in our work. When I think of self-determination, I think a lot about how if a service user doesn't want an intervention, I have to accept that, even though I may see that there's something we need to work on here. (Staff member 6)

Staff member 6 highlights the importance of respecting the service user's choices, even if they conflict with the staff's preferences. The staff member emphasises the value of the service user's right to self-determination, which agrees closely with the normalisation principle, which states that people with intellectual disabilities have the right to normal self-determination.

### 3.3 Barriers to an independent life

During the interview, staff were asked questions about what factors they perceive to be barriers to service users achieving an independent life. The results indicate that staff view the disability itself as a barrier. The disabilities identified by the staff included both physical impairments and intellectual disabilities.

Like our service user in a wheelchair. They don't have much independence outside their apartment … Because the service user can't just go out for a walk, if they want to go somewhere, we have to be there with them. (Staff member 2)

It varies greatly, so it's hard to give a concrete answer. Everyone has different conditions, so it depends on the person and what their challenges are … Generally speaking, it's common for them to have problems with time perception, which affects them a lot, like catching a bus and so on. It's also common for them not to understand the value of money. (Staff member 7)

The staff members' reasoning above reflects a medical model of thinking, which was the dominant perspective until the 1950s. None of the staff explained the challenges to independent living for the service users from a social perspective. As the quotes show, they all focused on the individual and the medical model.

### 3.4 Staff's experiences of the IRIS intervention

The interview material reveals that staff had varying experiences of the IRIS intervention. For instance, staff member 3 was unsure about the IRIS intervention, largely because they did not fully understand what should be done in IRIS. The interviewee mentioned that they are supposed to teach IRIS to the service users. This becomes challenging when they are unclear about what IRIS involves and how to implement it.

But we don't really know what it is we're supposed to do … We're already doing that kind of thing, so I don't think we really need IRIS for that … Yeah, we're supposed to be teaching it, but we don't know it ourselves, which feels a bit wrong. (Staff member 3)

During the interview, staff member 3 stated that they had requested more information about IRIS, because that they did not understand what it was and how they were supposed to use it. The interviewee expressed a wish that those responsible for IRIS would visit their workplace to provide more information about the intervention. Another interviewee (staff member 2) equated IRIS with PFA, stating that the working methods were similar. The interviewee does not have a negative attitude towards IRIS, and several staff members feel that IRIS is somewhat broader and deeper than PFA. According to the interviewee, IRIS is suitable for young people moving out of their family homes into their own apartments. With the help of IRIS, young people can become more independent and manage on their own.

…this is similar to PFA, where you observe them and see which tasks they can do on their own and which tasks are difficult. Then, based on that, you adjust, so it's a bit like PFA, I think. IRIS is perhaps a bit broader and it goes a bit deeper. I think it's probably intended for young people who are moving out and getting their own apartment. (Staff member 2)

In contrast to interviewees 2 and 3, the others were very positive about the IRIS intervention. They expressed that IRIS makes the work more concrete and provides a clearer structure. The interviewees highlighted the importance of IRIS being a collaborative project, which means that all the municipality's professionals are involved in the intervention in order to get a comprehensive understanding of the service user's needs.

I think it's a very good approach, of course we should be working towards the individuals' independence … overall, it's very good because you get a clear structure … that's my impression of IRIS. (Staff member 1)

…if I understand IRIS correctly, the idea is that we as case managers should all collaborate with the occupational therapist, physiotherapist, and so on. IRIS makes things concrete and sets time goals, these are things we work on all the time. Yes, it becomes more concrete when we actually set goals. (Staff member 7)

…IRIS itself is very good because I think we should work more intensively and for shorter periods … I felt that IRIS was very good and that it helps us to measure progress, yes, with the sub-goals. Have I improved? Was this step too easy? Can we take a bigger step? Was it too big? Was I unclear? I found it very instructive in that way … so I think IRIS was very good. (Staff member 4)

The above statements indicate that the staff feel the IRIS intervention works well with service users with disabilities. By breaking down goals into smaller sub-goals, it becomes easier for service users with disabilities to work towards a more independent life.

### 3.5 The impact of the IRIS intervention on the service users' support needs

The results show that the service users in the different residential homes have extensive support needs. These can include needing assistance with cooking, cleaning, and doing laundry. Some service users experience difficulties with these tasks due to (1) physical impairments, such as tremors, which make tasks like chopping onions and other ingredients more challenging. Some service users struggle with removing food from the oven, while others find it difficult to do laundry because they are unsure which items should be washed together and confused about which temperature to use. Some service users experience difficulties because of (2) inadequately adapted physical environments.

We are there to provide support with cooking, cleaning, and doing laundry … We started using IRIS … the physiotherapist and occupational therapist began working with the service user and noticed that the user had some tremors, which became an issue when they were chopping onions, for example. So, we tried a weighted vest, and it worked better. (Staff member 1)

Yes, the support need was for us to monitor the cooking. The service user wanted to do it independently. We had a bit of a preconceived idea that the service user could manage quite a lot, but we still needed to provide a bit more supervision. The service user often had to ask for help with tasks they couldn't manage … Taking food out of the oven was particularly difficult, so we discussed this together and eventually found a pair of very long oven mitts that went up to the elbows … that way, they could perform that part of the task independently. (Staff member 5)

The staff's statements above indicate that the service users were motivated to become more independent in various daily tasks such as cooking, cleaning, and doing laundry. However, they faced various difficulties in carrying out these tasks independently. With the help of the IRIS intervention, and in collaboration with other municipal professionals, the staff were able to support the service users to perform daily tasks independently by adapting the physical environment with items such as weighted vests, long oven mitts, and so on.

During the interviews, the staff were asked whether the IRIS intervention had helped the service users. The results show that most staff felt that IRIS has had a positive effect on the service users. They believed that the IRIS intervention had helped the service users become more independent, particularly with tasks like cooking and cleaning. The collaborative approach of IRIS, which involves staff from various professions within the municipality, has resulted in practical adjustments that have enabled the service users to perform daily tasks more independently, while also increasing their interest in these tasks.

Yes, it has definitely changed. The service user has moved from a group home to a supported housing unit. So, the project has been carried out, and the support needs that the service user had with us have essentially been ticked off. (Staff member 5)

### 3.6 The impact of IRIS on the service user's independence

Regarding the impact of IRIS on the service user's independence, the results show that some interviewees believed that the staff themselves negatively affect the service user's independence. This can happen when staff carry out various daily tasks for the service user—not because the service user is incapable of doing them, but because the staff can do them more quickly and thus save time.

Yes, but it's us, the staff, who are holding them back, precisely because we do things for them, and it's convenient … I told one service user, “You have to put on your clothes yourself”, and suddenly the user couldn’t do it. But I didn't give in. I stepped back and handed them the jumper, and they did it themselves. Admittedly, it took time—maybe half an hour. (Staff member 3)

Despite the tendency for staff to sometimes help service users more than necessary, the results show that the service users' independence has increased as a result of the IRIS intervention. According to staff, the service users have become more independent in their social interactions and are now more capable of booking transport via paratransit services for social engagements.

It was about the social side of things a bit. For example, the service user often didn't want to call friends to arrange a meet-up or call the paratransit company to book a taxi. But with some trained support interventions, like me making the call the first time, then us doing it together the next time, and building on that, the service user managed it. And by the end, they were booking paratransit independently and going to Ica Maxi on their own. The service user also independently booked trips to visit a friend who lives in another care home a bit further away. So, with the training we were able to gradually reduce the social anxiety. (Staff member 5).

People with intellectual disabilities are often regarded as non-independent. However, the above statements from the staff show that individuals with intellectual disabilities can achieve greater independence and autonomy. The ability to gain more independence and autonomy through the IRIS intervention can also improve their quality of life.

The staff's statements indicate that IRIS is appreciated not only by staff but also by service users. Staff member 5 highlights the importance of breaking down goals into smaller sub-goals to enable service users to achieve the set objectives. Collaboration with other professionals in the municipality is also emphasised as a crucial factor in working with the service users. Working on small sub-goals through IRIS helps to build the service users’ confidence.

There was a desire to move to supported housing, so the first thing we did was to make a mind map of what it would take to manage a home. Then we could break it down into smaller parts, and the service user and I could take one point at a time to work on and practise. This was part of IRIS, and we had good conversations between the professionals, which supported me with certain aspects of the physical training that was involved. (Staff member 5).

As highlighted by the staff, the fact that IRIS, helps to increase service users' confidence and enables them to perform everyday tasks more independently generates positive experiences and increases their belief in their own ability (self-efficacy).

### 3.7 Belief in one's own ability and a sense of empowerment

As previously mentioned, the staff perceive that the IRIS intervention helps service users live more independent lives by enabling them to perform certain daily tasks more independently. The results also show that the IRIS intervention can improve the service users’ self-esteem. During the interview, staff were asked how they perceived the service users' belief in their own abilities and sense of empowerment, both before and after the IRIS intervention.

Before IRIS, according to the staff, the service users had low self-confidence and little belief in their own abilities. The staff attributed this to the fact that other people around the service users (family, relatives, or staff) made most of the decisions for them and frequently told them that what they did or chose wasn't good enough.

The service user has often heard, “No, you can’t wear that shirt. Now you need to change your shirt”. “No, look at your hair, you can’t go out looking like that” … “No, no, I can’t decide what to wear”—things like that. Somewhere along the way, it affects them, and they start thinking, “Well, if I can’t even dress myself, maybe I can’t cook either”. It has something to do with self-confidence … We've become more aware of this since IRIS came into the picture. (Staff member 1)

I can see that the service user can do a lot more now than they ever thought they could before. Yesterday, the service user went to Ica Maxi on their own, bought groceries and products, and just taking that step is huge for them. Before, it was us, the staff, who had to be there to show them what to buy … now the service user goes and shops independently. (Staff member 5)

The above statements from the staff indicate that the service users' self-confidence, as well as their belief in their own abilities, were low before the IRIS intervention. The quotes clearly show that the service users have been positively affected by IRIS. Both their self-confidence and their belief in their abilities have been strengthened by achieving sub-goals that lead to the final objectives. Staff member 5's statements clearly show how the service user has become more independent because of the IRIS intervention and can manage daily tasks independently. In this case, the service user went to a shop and did the shopping on their own.

## 4 Discussion

The aim of this study was to investigate Intensive Rehabilitation Coordination (IRIS), a health-promoting and habilitative intervention that has the goal of preventing, reducing, and compensating for an individual's support needs. Allowing service users to be independent is a central and important principle in health and social care. This involves respecting and promoting the ability of individuals to make their own decisions, have control over their own life, and exercise self-determination within reasonable boundaries.

### 4.1 The concept of independence

From the interview material with staff, it became clear that there was no shared definition of independence in the workplaces, and that it was up to each person to define the concept. There were no common documents or seminars where staff could establish a shared understanding or create a foundation for a common definition, and this was not something that had been discussed at any staff meetings. In their definitions of independence, all the participants focused on the individual. Some believed that independence meant managing various daily tasks largely on one's own. Others viewed independence in terms of having control over one's life and making one's own choices, while some still defined independence in terms of possessing self-awareness, not relying on benefits, and not needing medication.

The lack of a common definition of independence may be one reason why different members of staff in care homes encouraged and supported service users in different ways. Some appeared to have a more restrictive interpretation, while others had a broader view. Those with a more restrictive interpretation believed that independence meant that the person must be able to perform all daily activities entirely on their own. Those with a broader view of independence sought to support the person in achieving their goals and performing activities as independently as possible, with or without assistance and adjustments. A consequence of not having a common definition was that staff had different ideas about what interventions were needed and what expectations should be met when providing care and support to residents with disabilities, which made it difficult to maintain a consistent and coordinated structure of care and support. According to (30), a clear and shared definition can promote consistency and efficiency in the workplace and ensure that rights are respected and laws are correctly followed. It can also promote better working relationships and create a more positive work environment for both staff and service users. To avoid confusion and conflict, and to ensure that the operation runs smoothly, clear definitions and goals are essential.

### 4.2 The impact of IRIS on the service user's life situation and independence

In response to the second research question—what opportunities can IRIS offer people with intellectual disabilities to experience increased autonomy and independence in different life situations—the results show that a clear majority of staff believe that the IRIS intervention has had a positive impact on the service users. According to the staff, IRIS has contributed to increasing the service users' independence, particularly in relation to everyday tasks such as cooking and cleaning. The IRIS intervention, which involves inter-professional collaboration within the municipality, has resulted in various measures that have promoted the service users' independence. Staff have been able to provide support and tools to enable the service users to carry out daily tasks independently. Furthermore, the IRIS intervention has increased the service users' interest and engagement in these tasks. This positive assessment of IRIS highlights the importance of inter-professional collaboration and individualised measures in care and support. Collaboration and the provision of appropriate support enabled service users to achieve a higher degree of independence and quality of life. The results suggest that staff perceive IRIS to have been successful in promoting the service users’ well-being and independence in their daily lives.

The staff noted that the service users expressed a strong desire to increase their independence in various daily tasks such as cooking, cleaning, and doing laundry. This is a positive sign, reflecting a desire to take more responsibility for their own daily lives. Despite this motivation, the service users encountered various obstacles when trying to carry out these tasks independently. To support the service users in achieving their desired level of independence, staff used the IRIS method and collaborated with other professionals in the municipality. By using small aids such as weighted vests and oven mitts, staff were able to provide the support required to enable service users to carry out daily tasks independently. The service users' progress and increased independence, particularly in performing daily tasks such as cooking, laundry, and cleaning, is consistent with Söder's (31) reasoning about the concept of independence. The staff's definition aligns closely with Söder's first definition of independence, namely the ability to perform tasks entirely independently, without assistance from others. The results of the IRIS intervention show that the service users have made progress in independence. Additionally, the results can be linked to the principle of normalisation (5), which emphasises that disabled people should have the opportunity to live as “normal” a life as possible. By offering support and tools that enable increased independence, the IRIS intervention contributed to this principle. Finally, the results also point to increased social inclusion for the service users, which agrees with Janson's (32) thoughts on social inclusion. By increasing the service users' independence and enabling them to participate in various life situations, the IRIS intervention fosters a more inclusive social environment for them. This suggests that IRIS not only has positive effects on an individual level but also contributes to broader societal benefits by promoting social inclusion and opportunities for people with disabilities to lead a “normal” life.

### 4.3 Challenges and areas for improvement in promoting independence

The results also show that some interviewees felt that the staff can have a negative impact on service users' independence. This happens when, for various reasons, the staff perform daily tasks for service users, even though the service users could manage them on their own. They do so not because the service users lack the ability to complete the tasks, but rather because they believe they can complete them more quickly or efficiently, which in their view saves time. This approach may reduce the service users' opportunities to practise and develop their skills, which in turn can limit their independence.

Despite the tendency of some staff to occasionally take over and do more than is necessary for the service users, the results from the staff interviews show that the IRIS intervention has had a positive impact on the service users' independence. According to the staff, the service users have become more independent in their interactions with friends and are better able to book transport for social engagements via paratransit services. It is common for people with intellectual disabilities to be seen as non-independent. However, the results show that people with such disabilities can achieve greater independence and autonomy with the help of the IRIS intervention.

The results also indicate that some service users, according to staff, have become more confident in expressing their opinions and wishes after receiving the IRIS intervention. This includes addressing issues such as cleaning and other household tasks. This increased independence and ability to communicate personal preferences may be a sign that the IRIS intervention has strengthened the service users' ability to take control of their lives and has boosted their self-confidence and belief in their ability to express their needs and wishes. This approach highlights the importance of tailoring support to each individual's specific needs and preferences. By using small but effective aids and collaborating with other professional actors, as recommended by the IRIS method, staff were able to promote the service users' independence and give them opportunities to take steps towards achieving their goals and desires in everyday life. The results are consistent with previous research showing the relationship between self-efficacy—the belief in one's ability to influence and manage different situations—and the experience of being able to control important aspects of one's own life (27, 28).

The staff emphasised the importance of breaking down overarching goals into smaller sub-goals and collaborating with other professionals in the municipality. By working on small sub-goals within the IRIS intervention, the service users gradually built up their self-confidence and finally achieved the overall goals. This approach is consistent with Bandura and Schunk's (33) theory on the value of encouraging individuals and creating achievable sub-goals for them as a way to counteract various forms of social exclusion. Bandura and Schunk (33) further state that when individuals successfully achieve different sub-goals, their motivation, self-confidence, and belief in their own abilities increase. This incremental approach to achieving independence has had a positive impact on the service users and their sense of self-confidence and independence through the IRIS intervention. This experience of positive change reflects the importance of small steps, as noted by Stajkovic and Luthans (34). According to Shantz and Latham (30), the opportunity to experience and achieve small successes can have a strengthening effect on the service users' self-esteem and increase their self-efficacy—their belief in their own ability to handle different situations and challenges.

## 5 Conclusion

The results of the study show that the IRIS intervention has had a positive impact on the service users' independence and increased their ability to manage various daily tasks, which has boosted their self-confidence and autonomy. By providing individualised support and collaborating with other professionals within the municipality, staff have been able to promote the service users' increased independence. This includes their ability to perform daily tasks such as cooking, cleaning, and doing laundry, as well as to express their opinions and preferences to a greater extent. The intervention has also promoted the social inclusion of people with disabilities and given them opportunities to lead a more “normal” life. However, there is some concern among staff that they may sometimes hinder the service users' independence by performing tasks for them rather than encouraging them to do them themselves. This underscores the importance of training and supporting staff to ensure that they understand and apply the principle of promoting service users' independence.

In summary, the findings indicate that staff perceive the IRIS intervention to have had a positive effect on the service users' independence and quality of life. Independence is conceptualised as a complex and dynamic experience, closely linked to the degree of self-determination and control that individuals have over their own life. A key insight that emerged from this study is the lack of a shared understanding of the concept of independence, which has resulted in staff adopting different approaches to their work. To enhance the future success and effectiveness of the IRIS intervention, it is crucial that both management and staff actively work to build consensus around the definition and meaning of independence. This will require them to agree on what independence entails within the framework of the IRIS intervention.

## Data Availability

The raw data supporting the conclusions of this article will be made available by the authors, without undue reservation.

## Footnotes

^1^The acronym LSS stands for the Swedish Act on Support and Services for Persons with Certain Functional Impairments (Lagen om stöd och service till vissa funktionshindrade).
